# Biochemical Markers of Saliva in Lung Cancer: Diagnostic and Prognostic Perspectives

**DOI:** 10.3390/diagnostics10040186

**Published:** 2020-03-27

**Authors:** Lyudmila V. Bel’skaya, Elena A. Sarf, Victor K. Kosenok, Ivan A. Gundyrev

**Affiliations:** 1 Laboratory of biochemistry, Omsk State Pedagogical University, 14, Tukhachevsky str, 644043 Omsk, Russia; nemcha@mail.ru; 2 Department of Oncology, Omsk State Medical University, 12, Lenina str, 644099 Omsk, Russia; victorkosenok@gmail.com; 3 LLC Bookmate, 7, Tsvetnoy bul’var str, 127051 Moscow, Russia; ivangundyrev@yandex.ru

**Keywords:** saliva, oncology, lung cancer, biochemical markers, diagnostics, prognostic markers

## Abstract

The aim of the work is to study the metabolic characteristics of saliva in lung cancer for use in early diagnosis and determining the prognosis of the disease. The patient group included 425 lung cancer patients, 168 patients with non-cancerous lung diseases, and 550 healthy volunteers. Saliva samples were collected from all participants in the experiment before treatment and 34 biochemical saliva parameters were determined. Participants were monitored for six years to assess survival rates. The statistical analysis was performed by means of Statistica 10.0 (StatSoft) program and R package (version 3.2.3). To construct the classifier, the Random Forest method was used; the classification quality was assessed using the cross-validation method. Prognostic factors were analyzed by multivariate analysis using Cox’s proportional hazard model in a backward step-wise fashion to adjust for potential confounding factors. A complex of metabolic changes occurring in saliva in lung cancer is described. Seven biochemical parameters were identified (catalase, triene conjugates, Schiff bases, pH, sialic acids, alkaline phosphatase, chlorides), which were used to construct the classifier. The sensitivity and specificity of the method were 69.5% and 87.5%, which is practically not inferior to the diagnostic characteristics of markers routinely used in the diagnosis of lung cancer. Significant independent factors in the poor prognosis of lung cancer are imidazole compounds (ICs) above 0.478 mmol/L and salivary lactate dehydrogenase activity below 545 U/L. Saliva has been shown to have great potential for the development of diagnostic and prognostic tests for lung cancer.

## 1. Introduction

Lung cancer is a malignant neoplasm that develops from the epithelial cells of the lung tissue. The largest number of deaths occurs in patients with lung cancer. In 2017, there were 2.2 million incident cases of lung cancer and 1.9 million death [1]. Such high rates are associated with untimely diagnosis; in particular, 41% of the detected cases of lung cancer in Russia in 2018 were in stage IV of the disease [2]. Both literature and our own experimental data show that lung cancer is usually detected at advanced stages, while the proportion of early cancer (T_1-2_N_0_M_0_) is 28% according to our data [3]. Survival analysis clearly demonstrates that overall survival is statistically significantly dependent on tumor size, the presence/absence of regional and distant metastases, while the dependence on the histological type of lung cancer is less pronounced [4].

Thus, in order to increase survival, it is critically important to diagnose lung cancer in the early stages, which, unfortunately, is not always possible with existing diagnostic methods [5]. To diagnose lung cancer, methods such as chest x-ray and sputum cytology have been tested and shown to be ineffective [6]. Currently, low-dose computed tomography of the chest is recommended for lung cancer screening, but its use is limited to the 55- to 74-year age group, and the target audience is heavy smokers or quitters of less than 15 years. High hopes are placed on the identification of early molecular markers of lung cancer (carcinoembryonic antigen (CEA), Cyfra 21-1, CA72-4 for adenocarcinoma; Cyfra 21-1, squamous cell carcinoma antigen (SCC)), CEA for squamous cell and large cell lung cancer; ProGRP, HCE, CEA for small cell lung cancer) [7]. However, the use of molecular markers is often limited to clarifying diagnostics, evaluating the effectiveness of treatment, predicting the course of the tumor process, and preclinical detection of relapse, and is used only in a number of cases for the active detection of cancer. Therefore, for the diagnosis of lung cancer, it is necessary to introduce new or expand the functionality of existing methods [8].

Problems of early diagnosis of lung cancer include the search for new tumor markers in blood plasma [9,10,11], sputum [12], and expired air [13,14,15,16]. They include mucins [17], antioxidant enzymes [18], microRNAs [19], fatty acids [20], cytokines [21], and so on. Several methodological approaches to interrogate liquid biopsies using circulating tumor cell (CTC) enumeration and characterization, transcriptomics, Raman spectroscopy, and copy number instability (CNI) scores using blood samples of lung cancer patients have been proposed [22,23]. An electronic nose (e-nose) is considered to be a promising technology that could be used to diagnose lung cancer. The e-nose can assess the volatile organic compounds (VOCs) detected in the breath and derived from the cellular metabolism (breathprint) [24]. However, there are few data from the literature on the study of the composition of saliva in lung cancer [25,26,27,28,29]. It was shown that profiling exosomal proteins in blood and saliva reveals more than 80% coincidence and can potentially be used to diagnose lung cancer [27]. Saliva microbiota may be an informative source for the detection of non-invasive lung cancer biomarkers [30]. In general, human saliva can be a good biological fluid for the early detection of lung cancer, because it can be collected non-invasively and contains a large amount of protein [9,31,32,33,34,35,36]. The exact mechanism by which markers of distal pathologies appear in saliva has not yet been determined. It has recently been discovered that small secretory lipid vesicles secreted by a tumor, called exosome-like micro vesicles (ELMs), can play a role in this phenomenon [37]. Two-dimensional gel electrophoresis with mass spectrometry was used to separate, quantify, and identify the salivary proteome. The combined effect of haptoglobin hp2, zinc-2-glycoprotein (AZGP-1), and calprotectin in saliva can reach 88% sensitivity and 92% specificity [38]. Recently, electric field-induced release and measurement (EFIRM) was introduced to detect the EGFR mutation in the saliva and plasma of patients with non-small cell lung cancer, and it was shown that an exon 19 deletion and L858R mutation can be detected [39,40]. It should be noted that at present none of the listed biomarkers are approved for use in clinical practice. Moreover, an integrated approach to the identification of biochemical markers of lung cancer in saliva has not yet been implemented, which makes research in this direction very promising.

This paper summarizes the results of a study of the metabolic characteristics of saliva in lung cancer, describes the prospects for using the results for the early diagnosis of lung cancer, and determines the prognosis of the disease.

## 2. Materials and Methods

### 2.1. Participants

This work is based on the results of examination and treatment of 593 patients hospitalized in the thoracic department of the Omsk Clinical Oncology Center during the period 2014–2017. The following criteria were considered as inclusion criteria: age of patients 30–75 years, and an absence of any treatment at the time of inclusion in the study including surgical, chemotherapeutic, or radiation. Patients were hospitalized for radical surgery in the scope of lobectomy, bilobectomy, pneumonectomy, combination treatment, or video thoracoscopy for tumor biopsy. In each case, histological verification of the diagnosis was performed. Saliva samples were collected strictly prior to treatment.

The structure of the study group is shown in Table 1. After histological verification, 168 people (28.5%) were diagnosed with non-cancerous lung pathologies, including: 51-hamartoma, 30-sarcoidosis, 28-tuberculoma, 39-fibrosis/pneumosclerosis, 13-inflammatory tumor, 4-pneumonia, 2-papilloma, 1-lipoma. These patients constituted a comparison group. In 425 patients, lung cancer of various histological types was confirmed, including: 189-adenocarcinoma (ADC), 135-squamous cell cancer (SCC), 8-mixed (ADC + SCC), 68-neuroendocrine cancer (NEC) and 25-undifferentiated lung cancer. The NEC group included 16 patients with a diagnosis of typical and atypical carcinoid (low grade G1 + G2), 45 patients with small cell lung cancer, and seven patients with large cell lung cancer (high grade G3). Additionally, the form of tumor growth was taken into account: 130-central cancer, 271-peripheral cancer, 17-mediastinal lung cancer, 7-without specification.

The control group included 550 conditionally healthy patients who did not reveal pulmonary pathology during routine medical examination. The Ethics Committee of the Omsk Regional Clinical Hospital “Clinical Oncology Center” approved the study on 21 July 2016 (Protocol No. 15).

### 2.2. Collection, Processing and Storage of Saliva Samples

Each patient collected unstimulated whole saliva in a volume of 5 mL on an empty stomach between 8:00 and 10:00 a.m. [41]. Subjects rinsed their mouth with water 10 min prior to sampling. The saliva samples were centrifuged (10,000× *g* for 10 min) (CLb-16, Moscow, Russia) [42]. The supernatant from each volunteer was divided into 29 aliquots. The biochemical parameters were determined immediately after centrifugation (without freezing).

### 2.3. Biochemical Analysis of Saliva Samples

The biochemical composition of the samples was established using the StatFax 3300 semi-automatic biochemical analyzer (Awareness Technology, Palm City, FL, USA) [43]. The pH, mineral composition (calcium, phosphorus, sodium, potassium, magnesium, chlorides), content of urea, total protein, albumin, uric acid, α-amino acids, imidazole compounds (ICs), seromucoids and sialic acids, activity of enzymes (aminotransferases (ALT, AST); alkaline phosphatase (ALP); lactate dehydrogenase (LDH); gamma-glutamyl transpeptidase (GGT); α-amylase) were determined in all samples. In all samples, the content of substrates for lipid peroxidation processes (diene conjugates, triene conjugates, Schiff bases, malondialdehyde (MDA)), and the level of middle molecules (MM) were determined [44]. Additionally, the activity of antioxidant enzymes (catalase, superoxide dismutase (SOD), peroxidase, total antioxidant activity (AOA)) was evaluated [45,46].

### 2.4. Statistical Analysis

The statistical analysis was performed by the Statistica 10.0 (StatSoft, Tulsa, OK, USA) program and R package (RStudio, version 3.2.3, Boston, MA, USA) while using the non-parametric method (Mann-Whitney U-criterion and Kruskal–Wallis test). The results are presented as the median (Me) and interquartile range in the form of the 25th and 75th percentiles. The differences were considered to be statistically significant at *p* < 0.05.

The survival curve was calculated by the Kaplan–Meier method and compared using the Log-rank test for univariate analysis (Statistica 10.0, StatSoft). Prognostic factors were analyzed by multivariate analysis using Cox’s proportional hazard model in a backward step-wise fashion to adjust for potential confounding factors. Overall survival (OS) was computed from the date of diagnosis to the date of death or the date of last follow-up. Survival data were obtained until December 2019.

The selection of parameters for constructing the classifier was carried out by a combination of several methods: ranking of parameters by importance (Rank Features By Importance), recursive exclusion of parameters (Recursive Feature Elimination), filtering of attributes based on various criteria (Information gain, Relief, Random Forest importance and others). Feature selection was carried out on the entire sample of 425 patients with lung cancer and 550 healthy volunteers. For the binary classification problem, a quality metric was used: the area under the ROC curve (Area Under Curve, Receiver Operator Characteristic (AUC-ROC)) [47]. Various classifiers were constructed on a set of basic images using informative parameters: linear discriminant analysis, naive Bayesian classifier, support vector method (SVM), gradient boosting (GBM), Random Forest, k-nearest neighbors method (kNN) [48], cross-validation (using the “caret” library). The best results for the AUC-ROC metric were obtained with the Random Forest classifier, with close results for GBM and SVM. This paper presents only the classification results using the Random Forest method.

## 3. Results

### 3.1. Metabolic Features of Saliva Composition in Patients with Lung Cancer

The results of biochemical analysis of saliva showed that its composition statistically significantly changes both against the background of non-malignant lung diseases and against the background of lung cancer (Table 2). Minimal changes are characteristic of the electrolyte composition of saliva, namely: with lung cancer, the level of calcium, potassium, and chloride ions increase with statistical significance. The maximum changes were noted for the parameters of lipid peroxidation and protein metabolism, which generally corresponds to the overall picture of metabolic changes occurring against the background of cancer.

To confirm the hypothesis that the revealed changes were due to the presence of an oncological disease, the listed biochemical parameters were evaluated against the background of non-cancerous tumor pathology (comparison group). It was shown that during the transition from the control group to the comparison group, and then to the main group, the following occurred: the protein content decreased (−15.0% and −18.8%); the content of triene conjugates (+12.5% and +2.4%) and Schiff bases (+5.7% and +5.5%) increased, as did the content of the final lipoperoxidation product, MDA (+7.5% and +5.0%, respectively). There was also a decrease in the content of the fraction of middle molecules MM 254 nm (−3.7% and −7.0%) and an increase in the distribution coefficient of MM 280/254 nm, which reflects the rate of accumulation of low molecular weight proteins and peptides (+6.3% and +5,9% respectively). The nature of the change in the studied parameters is ambiguous and depends both on the histological type of the tumor and on the stage of the disease, including the presence/absence of distant and regional metastasis (Supplementary Tables S1–S3).

Violation of protein metabolism was manifested in an increase in the content of α-amino acids (+2.2% and +1.0%), imidazole compounds (+29.5% and +10.7%), and seromucoids (+12.2% and +8.9%) against the background of a decrease in protein content (−15.0% and −18.8%) and sialic acids (−21.5% and −12.3%, respectively).

It has been shown that, in lung cancer, oxidative stress develops, which is accompanied by an imbalance in the antioxidant defense of saliva. Thus, the activity of catalase is significantly reduced (−31.9% and −38.0%), while the activity of salivary peroxidases increases (+68.8% and +37.5%, respectively). Non-enzymatic protection indicators change in different directions: the level of uric acid decreases with pathologies of the lungs (−7.9% and −3.7%), while the concentration of albumin increases (+19.2% and +15.4%, respectively) and, under these conditions, starts to exhibit prooxidant properties.

Against the background of lung cancer, a change in the activity of metabolic enzymes was observed. A statistically significant increase in ALT activity was established (+1.9% and +10.8%) with a slight decrease in AST activity and AST/ALT coefficient decreases (−4.2% and −12.0%, respectively, *p* < 0.05). The activity of ALP in the transition from the control group to the comparison group and main one increased (+22.2% and +25.9%), while the activity of GGT increased only for the main group (+7.4%). A decrease in the activity of the studied enzymes against the background of disease progression was shown, including the presence of distant and regional metastasis (Supplementary Tables S1–S3). The activity of salivary α-amylase was higher both for the comparison group and the main group (+55.0%).

### 3.2. Diagnostic Capabilities of Saliva for Lung Cancer

According to the data given in Table 2, the biochemical composition of saliva against the background of lung cancer varies significantly; however, none of the determined biochemical parameters can be used to diagnose lung cancer independently.

**Figure 1:**
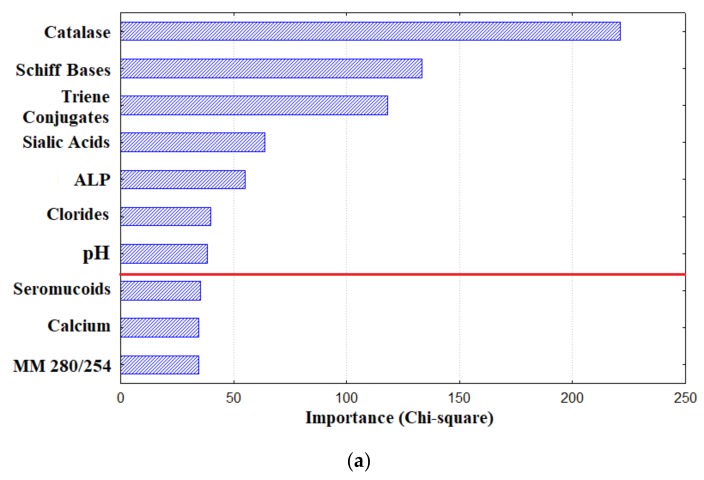

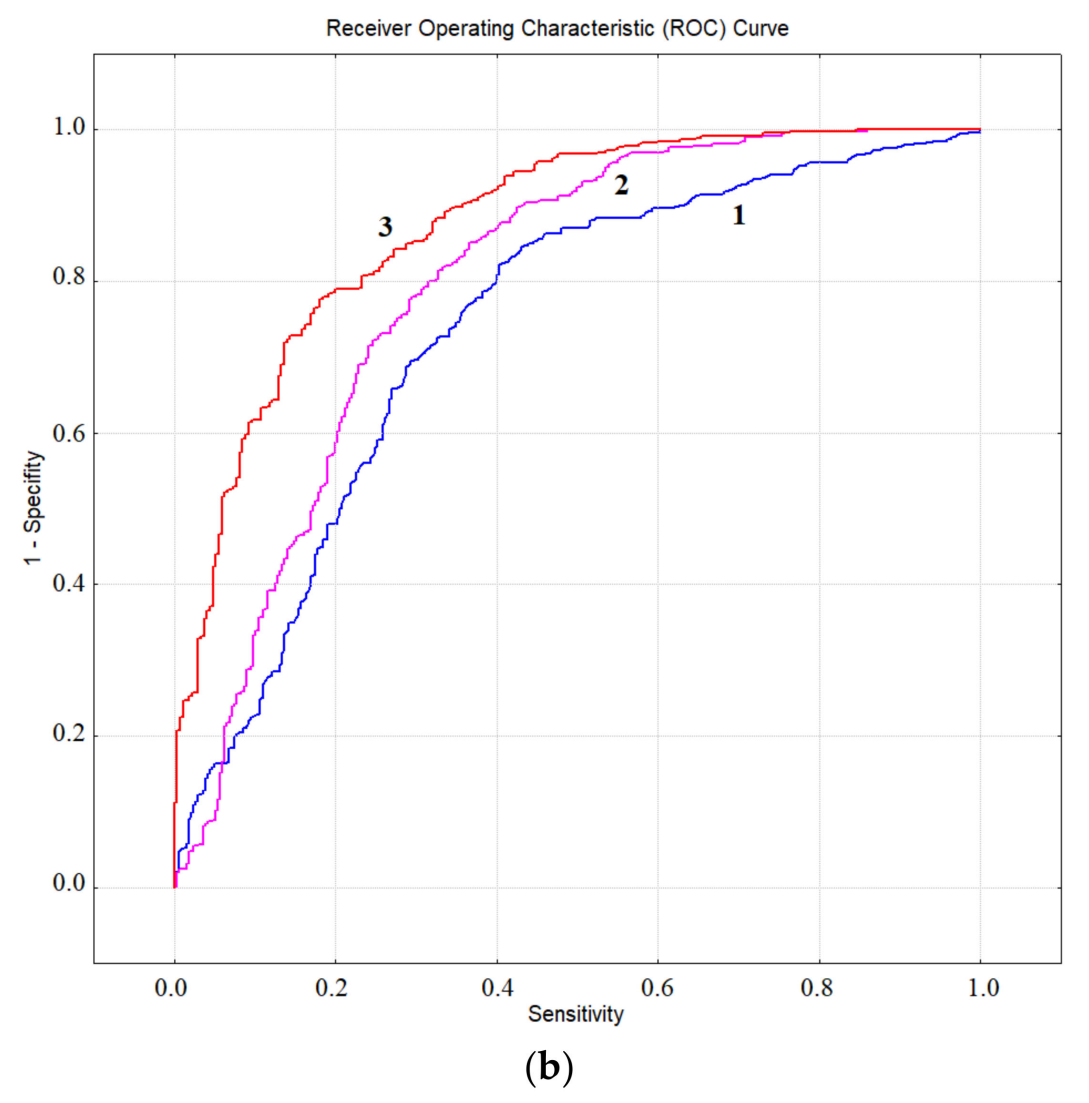
Selection of features for constructing a classifier: (**a**) the result of sorting the biochemical parameters of saliva by importance (the first 10 parameters are given); the red line indicates the parameters included in the classifier; (**b**) the sensitivity and specificity of the diagnosis when using only catalase as features (curve 1, AUC ROC = 0.738), the first three features selected earlier (curve 2, AUC ROC = 0.764), and all seven features used later to construct the classifier (curve 3, AUC ROC = 0.803).

The next step was the selection of the most informative parameters (features), which included catalase activity, the level of triene conjugates and Schiff bases, pH, sialic acid concentration, alkaline phosphatase activity, and chloride ion content (Figure 1a). The calculation of sensitivity and specificity showed that the maximum values corresponded to the activity of catalase in saliva and were at a level of 70%. As the number of features increased, the accuracy increased and reached values of about 80% when seven parameters were taken into account (Figure 1b). A further increase in the number of features did not significantly affect the diagnostic characteristics of the method, but significantly increased its complexity.

Additionally, traits were selected separately for each histological type of lung cancer (Supplementary Figures S1–S3). It has been established that the same features were selected as the most important ones for non-small cell lung cancer (ADC and SCC), while for neuroendocrine lung cancer, pH was added instead of sialic acids (Supplementary Figures S1–S3).

**Figure 2:**
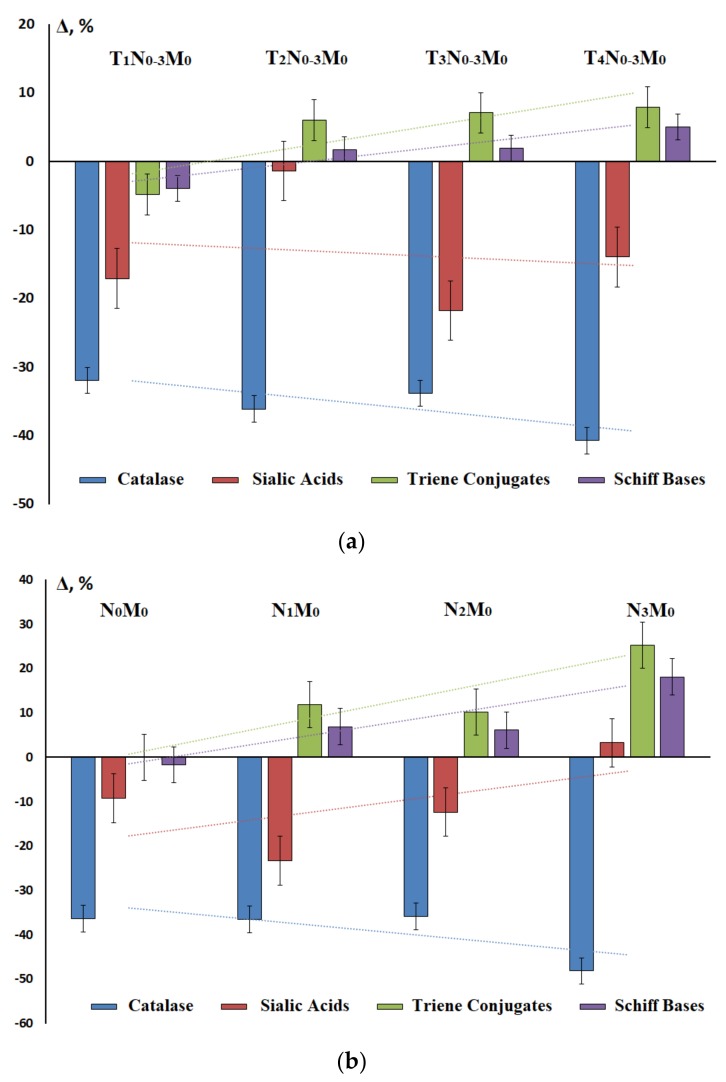
Changes in catalase activity, sialic acid levels, triene conjugates, and Schiff bases depending on: (**a**) tumor size (T) and number of patients in groups T_1_N_0__-3_M_0_ – 30, T_2_N_0__-3_M_0_ – 153, T_3_N_0–3_M_0_ – 72, and T_4_N_0-3_M_0_ – 54 (Supplementary Table S2); (**b**) from the presence/absence of the lymph node metastasis (N) and number of patients in groups N_0_M_0_ – 146, N_1_M_0_ – 59, N_2_M_0_ – 88, and N_3_M_0_ – 16 (Supplementary Table S3).

The choice of these parameters was quite logical, since we previously showed that they statistically change significantly with the progression of the disease, and therefore can be used as diagnostic criteria (Figure 2a,b). It was shown that with the progression of the disease there was an increase in lipid peroxidation, while the activity of antioxidant enzymes, and catalase in particular, decreased. In the same direction, the level of sialic acids decreased (Figure 2a). In the presence of regional metastasis, the same trend remained; however, the level of sialic acids increased slightly with an increase in the number of metastases in the lymph nodes (Figure 2b). A detailed description of the changes in all the studied parameters depending on the stage of the disease and presence/absence of distant and regional metastasis is given in Supplementary Tables S2 and S3.

Next, a model classifier for the diagnosis of lung cancer was constructed, based on the values of seven biochemical parameters of saliva using the Random Forest method. The values of sensitivity and specificity (69.5% and 87.5%, respectively) were estimated by cross-validation. The given values of sensitivity and specificity were averaged for lung cancer at various stages. For the developed model classifier, an analysis was made of the data of patients who were erroneously classified according to the stage of the disease (Table 3). It was shown that the maximum number of classifier errors corresponded to the early stages (T_1-2_N_0_M_0_), for which metabolic changes were not very pronounced, as well as advanced stages (T_1-4_N_0-3_M_1_) of lung cancer, for which a change in biochemical parameters can be distorted by corresponding changes in organs that are affected by metastases of lung cancer. This pattern may also be associated with a large number of patients in the early and advanced stages compared to other groups. In general, stable diagnostic characteristics of the classifier at all stages of the disease should be noted (Table 3).

### 3.3. Prognostic Value of Biochemical Markers of Lung Cancer in Saliva

**Figure 3:**
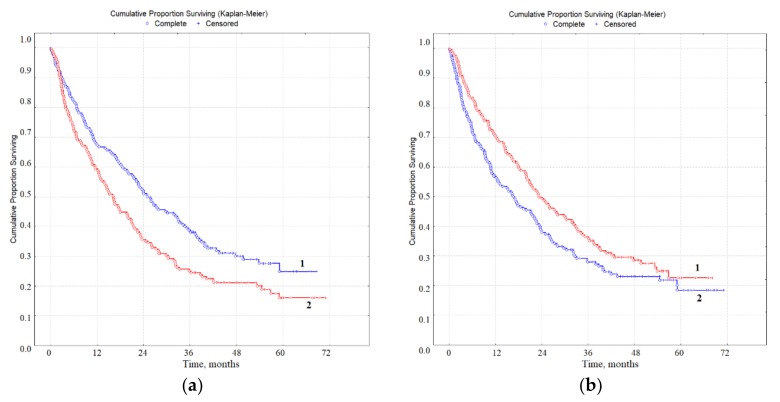

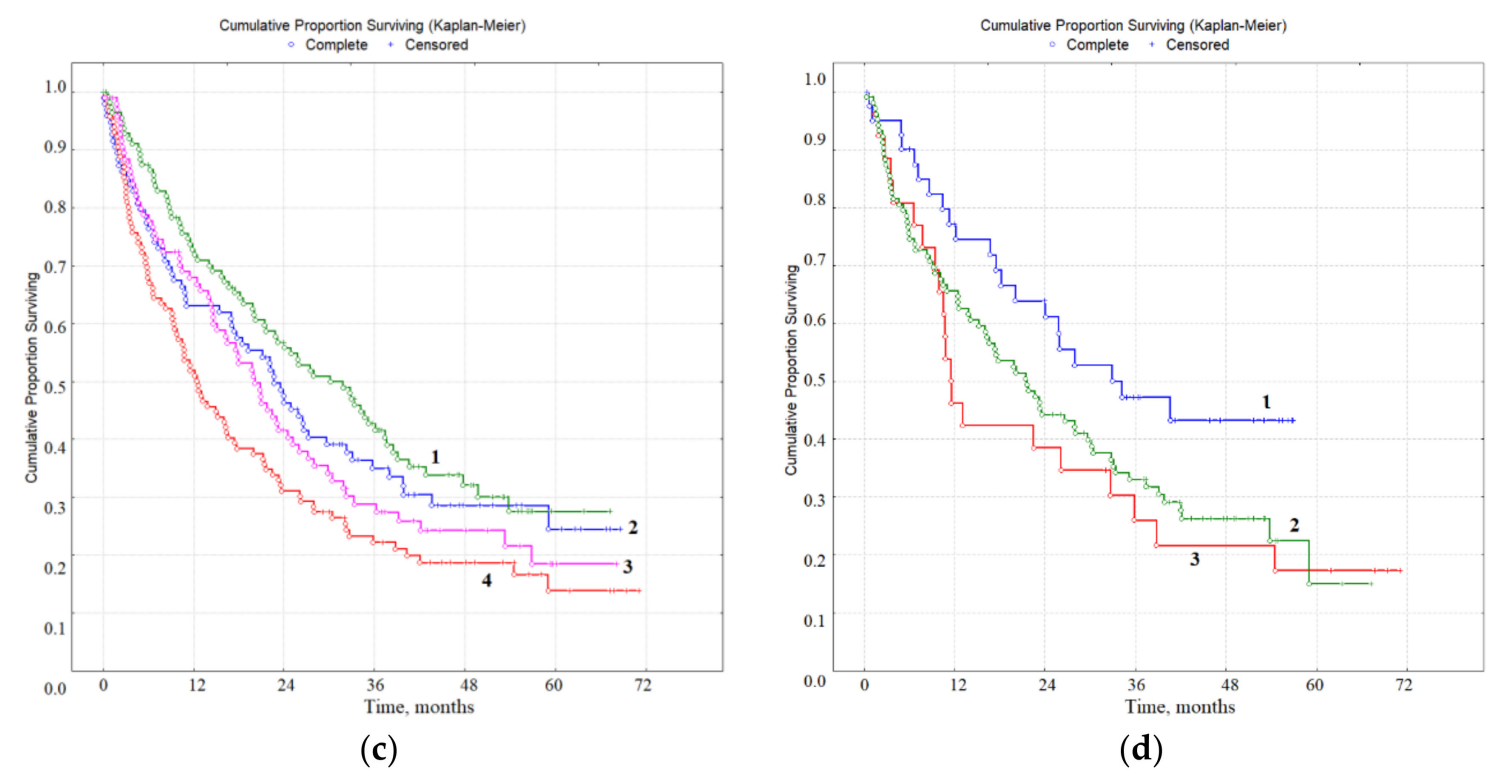
The overall survival of patients with lung cancer, depending on the level of IС and LDH activity in saliva: (**a**) IC < 0.311 mmol/L (curve 1) and IC > 0.311 mmol/L (curve 2); (**b**) LDH > 1133 U/L (curve 1) and LDH < 1133 U/L (curve 2). (**c**) Multivariate regression analysis of overall survival: IC < 0.311 mmol/L and LDH > 1133 U/L (curve 1), IC < 0.311 mmol/L and LDH < 1133 U/L (curve 2), IC > 0.311 mmol/L and LDH > 1133 U/L (curve 3), IC > 0.311 mmol/L and LDH < 1133 U/L (curve 4). (**d**) Concentration values of IC and LDH vary relative to the interquartile range: IC < 0.197 mmol/L and LDH > 1748 U/L (curve 1), 0.197 < IC < 0.478 mmol/L and 545 < LDH < 1748 U/L (curve 2), IC > 0.478 mmol/L and LDH < 545 U/L (curve 3).

According to the results of multivariate regression analysis, it was found that the concentration of imidazole compounds (ICs) and the activity of lactate dehydrogenase (LDH) of saliva were significantly associated with survival rates for patients with lung cancer (IC, *p* = 0.0033; LDH, *p* = 0.0057). Since the medians of concentration were 0.311 (0.197; 0.478) mmol/L for IC, 1133 (545.5; 1478.9) U/L for LDH (Table 2), these values were used as threshold values in the assessment of overall survival (Table 4, Figure 3). An IC level of less than 0.311 mmol/L and LDH activity of more than 1133 U/L were predictively favorable (Figure 3a,b). The combination of both parameters was a more effective prognostic sign (Table 4). For patients with a favorable prognosis (IC < 0.311 mmol/L and LDH>1133 U/L) one, three, and five years survival were 1.4, 1.9, and 2.0 times higher than for patients with a poor prognosis (IC < 0.311 mmol/L, LDH < 1133 U/L) (Figure 3c).

Since the range of variation of the studied parameters was quite wide, in the next stage separation was carried out in accordance with the interquartile range: IC < 0.197 mmol/L and LDH < 545 U/L; IC 0.197–0.478 mmol/L and LDH 545–1748 U/L; and IC > 0.478 mmol/L and LDH > 1748 U/L (Table 4). Only three combinations of parameters are shown in Figure 3d, and it can be seen that the differences between groups with a favorable and unfavorable prognosis were statistically significant (*p* = 0.0040). The one-year survival rates sharply decreased with an unfavorable prognosis from 77.0% to 46.8%, the three-year survival decreased from 47.5% to 27.1%, while the five-year survival decreased from 43.3% to 18.0% (Figure 3d). The combination of intermediate values in terms of survival was closer for the group with an unfavorable prognosis, but the differences between groups were not statistically significant.

## 4. Discussion

In the course of the study, it was shown that saliva can be used as a promising biological fluid to detect metabolic changes in the presence of cancer, and lung cancer in particular. Earlier in the literature, metabolic changes occurring specifically in saliva in lung cancer were not described. Thus, it has been shown that in lung cancer, oxidative stress develops, which manifests itself as an increase in the level of lipid peroxidation products and endogenous intoxication in saliva. The pathogenetic role of oxygen free radicals and the processes of lipid peroxidation initiated by them in the development of diseases, including oncological ones, is widely known [49,50,51,52]. Oxidative stress s manifests as an accumulation of damaged DNA bases, products of protein oxidation, and lipid peroxidation, as well as a decrease in the level of antioxidants and the associated increased susceptibility of membrane lipids and lipoproteins to the action of prooxidants [53,54]. In the lungs, oxidative stress induces protein modification, macrophage activation, and neutrophil recruitment in the central and peripheral airways; the accumulation of toxic products of lipid peroxidation, hydrogen peroxide, nitrosothiols, and nitrates in the membranes of the lungs and blood; as well as in exhaled air [55,56,57,58,59]. In addition, oxidative stress can provoke hyperplasia of the mucous membranes of the glands and apoptosis of epithelial cells of the bronchi [60]. Complex metabolic disorders and nonspecific clinical manifestations that accompany the development of malignant neoplasms are characterized as endogenous intoxication syndrome [61,62,63]. An increase in the ratio of MM 280/254 nm is indirect evidence of the excessive generation of active oxygen metabolites, superoxide radicals, and hydrogen peroxide [64]. Hydroxyl radicals are capable of damaging the phosphoglyceride membrane structures of cell membranes and their organoids. The object of exposure to active oxygen metabolites is arachidonic acid containing four double bonds separated by CH_2_ groups. When exposed to hydroxyl radicals, double bonds become conjugated and diene conjugates are formed, which later turn into lipid hydroperoxides. It was shown that the level of diene conjugates increased with lung cancer compared to a control group [65,66]. The level of secondary products against the background of lung cancer rose regardless of the histological type of tumor [67,68] (Supplementary Table S1). Increasing the level of Schiff bases is an adaptive process aimed to eliminate more toxic metabolite MDA from the cells. Based on the assumption that the primary product of MM formation is acyl hydroperoxides and fragments of damaged cell membranes, an equilibrium shift towards the accumulation of lipoperoxidation products was observed, and the processes of endogenous proteolysis due to lung cancer was slowed down. It should be noted that the content of MDA is higher in lung cancer; however, a statistically significant increase in this indicator could not be detected, despite numerous confirmations in literature [69,70,71,72,73,74,75,76]. It is known that the main factors in the formation of oxidative stress are active forms of oxygen and nitrogen, which are highly reactive and cause, in particular, oxidative modification of biopolymers (proteins, lipids, nucleic acids, carbohydrates), which ultimately lead to tissue respiration in the internal mitochondrial membrane and hydroxylation processes in microsomes [77]. The system of inhibition of excessive auto oxidation consists of non-enzymatic and enzymatic units [78]. Specific antioxidant enzymes include superoxide dismutase (SOD), catalase, glutathione peroxidase, glutathione reductase, and transferase [79,80]. This group of enzymes, localized mainly intracellularly, has the ability to destroy free radicals, as well as to participate in the decomposition of hydroperoxides in a non-radical way. Among non-enzymatic antioxidants, uric acid, ascorbate, and albumin can be distinguished that can intercept excessively produced free radicals [81,82]. In the course of our studies, a statistically significant decrease in the antioxidant defense of saliva has been established, and this is reflected in both the enzymatic and non-enzymatic units (Table 2) [46]. It has been shown that a statistically significant decrease in catalase activity was observed both in the main group and in the comparison group [45].

Against the background of lung cancer, metabolic changes were observed, characterized by a decrease in the de Ritis coefficient due to an increase in the activity of alanine aminotransferase against the background of an increase in the activity of gamma-glutamyltransferase and alkaline phosphatase, as well as a decrease in the activity of lactate dehydrogenase (Table 2) [83]. Gamma-glutamyltransferase is an enzyme responsible for the transport of amino acids into cells, and an increase in the activity of gamma-glutamyltransferase enhances the flow of amino acids through the cell membrane. A decrease in lactate dehydrogenase activity means a general inhibition of energy systems. It is known that in the blood of lung cancer patients the activity of NAD- and NADP-dependent dehydrogenases (including lactate dehydrogenase) is reduced, which means a decrease in the intensity of anaerobic and aerobic energy processes [84]. An increase in the activity of alanine aminotransferase can also be considered an increase in the role of the alanine glucose pathway, with the release of glucose from cells due to its dephosphorylation with high alkaline phosphatase activity [85,86]. Alkaline phosphatase is involved in transmembrane phosphorylation, providing, along with the hormonal system, the entry and exit of glucose into the cells, which directly affects the level of glucose in the blood and plays a role in maintaining the level of phosphates needed for bioenergy. In this regard, inhibition of the final pathways of glucose metabolism was observed, as evidenced by the low activity of aspartate aminotransferase, which is involved in a decrease in the de Ritis coefficient. Such changes in enzymatic activity may reflect the stimulation of peripheral metabolic zones, especially protein, against the background of inhibition of central metabolic pathways.

It was shown that the normal content of sialic acids was higher than with lung pathologies, while the concentration of imidazole substances and seroglycoids was significantly lower [87]. Imidazole derivatives include the amino acid histidine and its metabolites (histamine, urocanic acid, etc.). The processes of malignancy and malignant growth cause significant changes in histidine catabolism. As a result of intramolecular deamination from histidine under the action of the enzymes histidase and urocaninase, urocanic acid is formed. It is known that in malignant tumors of various localizations, a decrease in the synthesis of enzymes occurs until its suppression is almost complete [88]. In this regard, the synthesis of urocanic acid against the background of the neoplastic process is also suppressed. However, the level of endogenous histamine increases both in the blood plasma and in the tumor tissue itself [89]. Evidence has been obtained for the secretion of histamine by the tumor cells, as well as of the histamine metabolizing enzyme histaminase [90]. It has been suggested that an increase in the activity of histaminase in a tumor contributes to a change in the metabolism of polyamines and the formation of reactive oxygen species involved in carcinogenesis [91]. Histamine is involved in the processes of inflammation and repair, increasing vascular permeability, triggering the cytokine cascade, and the activation of immune cells, stimulating angiogenesis. In oncogenesis, histamine can stimulate the proliferation and angiogenesis of a tumor, increasing its growth rate [92]. It is believed that with neoplastic pathology, including lung cancer, the level of histamine is a parameter for monitoring the disease [87].

It is necessary to clarify that the objectives of this study did not include the development of a diagnostic method that demonstrates higher diagnostic characteristics compared to existing ones. The model classifier was chosen as an example, because current clinical recommendations for the diagnosis of lung cancer do not include a single method based on a multivariate assessment of laboratory test results [47]. Nevertheless, when compared with existing markers of lung cancer, the sensitivity and specificity for Cyfra 21-1 were 43% and 89%, for CEA they were 57% and 92%, for SCC they were 75% and 90%, and for EGFR they were 71% and 80%, respectively. Markers of small cell lung cancer showed sensitivity and specificity of 23% and 98% for HCE, and 78% and 95% for ProGRP [9]. Modern requirements for markers of cancer are becoming more stringent, and to reduce the number of false-positive results it is necessary to increase specificity, bringing it as close as possible to 100% [93]. In this regard, all existing markers are not good enough. Nevertheless, the obtained values of sensitivity and specificity of 69.5% and 87.5% are a good basis for the search for new biochemical markers in saliva. It is also possible to develop a classifier that combines the parameters proposed in this work and standard markers (CEA, Cyfra 21-1, HCE, etc.), but in this case it is necessary to develop a classifier, taking into account the histological type of lung cancer, which is planned to be done at the next stage of work. Even if the applicability for early diagnosis is not proven, it is possible to use salivary biomarkers to check the effectiveness of the therapy, monitor the course of the disease, identify residual tumors, predict the clinical course, and select an effective therapeutic practice [94].

The literature also does not mention the use of biochemical saliva markers for prognostic purposes in lung cancer. However, it has been shown for blood plasma that prognostic ones may be AFR (the albumin/fibrinogen ratio) for non-small cell lung cancer (NSCLC) [95], LDH for both NSCLC and SCLC [96,97], AAPR (albumin/alkaline phosphatase ratio) [98], the level of Cyfra 21-1 [99], the content of selenium [100], among others. In particular, an increased AFR value is prognostically favorable, and patients with a higher AFR have had a lower risk of death (HR = 0.512, *p* = 0.006) [95]. Multivariate analysis showed that lactatedehydrogenase B (LDHB) expression was an independent risk factor in lung SCC (HR = 0.393, *p* = 0.028). A positive correlation was found between LDHB expression and serum LDH level (*p* = 0.02). High LDHB expression is significantly associated with the level of serum LDH and better clinical outcomes in lung SCC [96]. In both limited and extensive disease SCLC, elevated LDH serum levels resulted in significantly shorter median survival. The effect was most pronounced if levels were 300 U/L or higher. In patients with limited disease and normal LDH levels, median survival was 18.0 months. If LDH was higher than 300 U/L, overall survival was reduced to 12 months [97]. Patients with AAPR < 0.57 had significantly lower rates of OS and disease free survival (DFS) than those of patients with AAPR > 0.57 (*p* < 0.001). These differences remained significant after subgroup analyses and PSM analyses. Multivariate analyses on the entire cohort and the PSM cohort commonly indicated that low preoperative AAPR could be an independent prognostic factor for unfavorable OS and DFS of resected NSCLCs [98]. In a multivariate analysis using the variables found to be significant prognostic factors in univariate analysis, a high Cyfra 21-1 level was found to be a significant independent prognostic factor (95% confidence interval 1.213–5.442, *p* = 0.014) [99]. In patients undergoing treatment for stage I lung cancer, serum selenium levels at the time of diagnosis (>69 μg/L) may be associated with improved overall survival [100].

We have shown that LDH activity and level of imidazole compounds are prognostically important biochemical markers in saliva. It should be noted that, in contrast to the previously obtained data for blood plasma, a prognostically favorable sign is an increased level of LDH of saliva before treatment (Table 4). This fact is explainable, since a correlation between the LDH activity of blood plasma and saliva has not been identified, and therefore the values of these indicators in saliva should be considered as a separate diagnostic indicator for which it is necessary to establish criteria of norm and pathology [101]. Data on their prognostic value in lung cancer have not been obtained previously for imidazole compounds; a decrease in the level of imidazole compounds in saliva is prognostically favorable (Table 4). In general, a combination of both parameters in the case of a favorable prognosis corresponds to a median of overall survival of 22.4 months, while in the case of an unfavorable prognosis, it is two times less (11.7 months). These indicators can be used to predict the course of the disease and adjust patient treatment tactics.

The limitations of the study include the construction of a classifier for the diagnosis of lung cancer without taking into account the histological type of tumor, as well as the determination of prognostic factors without taking into account the type of treatment and the stage of the disease. These shortcomings are planned to be eliminated at the next stage of the study.

## 5. Conclusions

Saliva has been shown to have great potential for the development of diagnostic and prognostic tests for lung cancer. Complex of metabolic changes occurring in saliva against lung cancer were described. Seven biochemical parameters (catalase activity, level of triene conjugates and Schiff bases, pH, sialic acid concentration, alkaline phosphatase activity, and chloride ion content) were identified and used to construct the classifier. The diagnostic characteristics of the developed classifier were 69.5% and 87.5% for sensitivity and specificity, respectively, which is practically not inferior to the diagnostic characteristics of markers routinely used in the diagnosis of lung cancer. Significant independent factors in the poor prognosis of lung cancer were imidazole compounds above 0.478 mmol/L and salivary lactate dehydrogenase activity below 545 U/L. These indicators can be used to predict the course of the disease and adjust patient treatment tactics.

## Figures and Tables

**Table 1:** The structure of the study group.

Feature	Lung Cancer, *n* (%)			Non-Malignant Lung Diseases, *n* = 168
	ADC, *n* = 189	SCC, *n* = 135	NEC, *n* = 68	
**Age**, **Years**	61.0 [56.0; 65.0]	59.0 [55.0; 66.5]	55.0 [52.0; 60.0]	55.0 [45.5; 60.5]
**Gender**				
Male	129 (68.3)	128 (94.8)	50 (73.5)	98 (58.3)
Female	60 (31.7)	7 (5.2)	18 (26.5)	70 (41.7)
**pT**				
T1	21 (11.1)	4 (3.0)	5 (7.3)	-
T2	105 (55.6)	47 (34.8)	31 (45.6)	-
T3	30 (15.9)	53 (39.3)	8 (11.8)	-
T4	33 (17.4)	31 (22.9)	24 (35.3)	-
**pN**				
N0	82 (43.4)	51 (37.8)	19 (27.9)	-
N1	34 (18.0)	34 (25.2)	13 (19.2)	-
N2	51 (27.0)	45 (33.3)	24 (35.3)	-
N3	22 (11.6)	5 (3.7)	12 (17.6)	-
**pM**				
M0	133 (70.4)	108 (80.0)	48 (70.6)	-
M1	56 (29.6)	27 (20.0)	20 (29.4)	-

ADC: adenocarcinoma; SCC: squamous cell carcinoma; NEC: neuroendocrine cancer; pT, pN and pM: stages according to UICC TNM staging system (7th edition).

**Table 2:** The results of biochemical analysis of saliva.

Indicators	Control Group	Comparison Group	Lung Cancer	Kruskal–Wallis test (H, *p*)
**Electrolytes**				
pH	6.50 [6.30; 6.72]	6.50 [6.25; 6.82]	6.49 [6.23; 6.79]	0.5756, 0.7500
Calcium, mmol/L	1.33 [1.05; 1.66]	1.38 [1.02; 1.75]	1.42 [1.00; 1.85]	4.6847, 0.0961
	-	-	*p_1-3_* = 0.0306	
Phosphorus, mmol/L	4.53 [3.58; 5.85]	4.73 [3.25; 5.65]	4.56 [3.37; 5.77]	1.9933, 0.3691
Sodium, mmol/L	8.4 [5.5; 12.4]	8.2 [5.6; 11.1]	9.1 [5.8; 14.6]	3.7291, 0.1550
Potassium, mmol/L	11.8 [9.3; 14.7]	12.0 [8.7; 14.8]	12.9 [9.5; 16.3]	7.6743, 0.0216
	-	*p_1-2_* = 0.0352	*p_1-3_* = 0.0118	
Chlorides, mmol/L	26.1 [21.2; 32.2]	23.4 [18.1; 31.1]	28.3 [21.7; 36.3]	18.5965, 0.00009
	-	*p_1-2_* = 0.0012	*p_1-3_* = 0.0059	
Magnesium, mmol/L	0.300 [0.246; 0.350]	0.293 [0.223; 0.366]	0.300 [0.230; 0.372]	1.4687, 0.4798
**Nitric oxide (NO)**, nmol/mL	23.5 [13.5; 38.1]	21.7 [11.9; 37.0]	24.0 [14.2; 42.3]	1.5079, 0.4705
**Protein Metabolism**				
Protein, g/L	0.80 [0.50; 1.23]	0.68 [0.48; 1.00]	0.65 [0.34; 1.04]	20.6393, 0.00003
	-	*p_1-2_* = 0.0140	*p_1-3_* = 0.0000	
Albumin, g/L	0.26 [0.17; 0.44]	0.31 [0.17; 0.51]	0.30 [0.16; 0.48]	3.937, 0.1397
	-	*p_1-2_* = 0.0121	-	
Urea, mmol/L	7.84 [5.40; 11.03]	7.44 [4.93; 10.31]	8.00 [5.76; 11.86]	5.8911, 0.0526
	-	*p_1-2_* = 0.0001	-	
Uric acid, nmol/mL	86.49 [28.18; 154.77]	79.64 [32.18; 151.38]	83.33 [36.54; 166.67]	1.8995, 0.3868
α-amino acids, mmol/L	4.12 [3.83; 4.50]	4.21 [3.94; 4.57]	4.16 [3.88; 4.61]	5.4938, 0.0641
	-	*p_1-2_* = 0.0487	*p_1-3_* = 0.0270	
Imidazole compounds (ICs), mmol/L	0.281 [0.175; 0.379]	0.364 [0.220; 0.501]	0.311 [0.197; 0.478]	34.1462, 0.00000
	-	*p_1-2_* = 0.0000	*p_1-3_* = 0.0000	
Seromucoids, c.u.	0.090 [0.060; 0.130]	0.101 [0.062; 0.147]	0.098 [0.055; 0.154]	1.5811, 0.4536
Sialic acids, mmol/L	0.195 [0.134; 0.299]	0.153 [0.088; 0.211]	0.171 [0.095; 0.281]	36.2003, 0.00000
	-	*p_1-2_* = 0.0000	*p_1-3_* = 0.0000	
**Metabolic enzymes**				
**Alanine aminotransferase** (ALT), U/L	3.62 [2.54; 4.92]	3.69 [2.77; 5.31]	4.01 [2.77; 5.62]	13.3327, 0.0013
	-	*p_1-2_* = 0.0035	*p_1-3_* = 0.0003	
**Aspartate aminotransferase** (AST), U/L	5.50 [3.67; 7.33]	5.42 [3.58; 7.75]	5.25 [3.17; 7.50]	1.6532, 0.4375
AST/ALT	1.42 [1.13; 1.92]	1.36 [1.01; 1.79]	1.25 [0.96; 1.60]	34.349, 0.00000
	-	*p_1-2_* = 0.0026	*p_1-3_* = 0.0000	
**Alkaline phosphatase** (ALP), U/L	58.67 [41.29; 82.57]	71.71 [52.15; 117.34]	73.88 [49.98; 117.34]	46.515, 0.00000
	-	*p_1-2_* = 0.0000	*p_1-3_* = 0.0000	
**Lactate dehydrogenase** (LDH), U/L	1127.5 [652.1; 1838.0]	1140.0 [541.5; 1802.0]	1133.0 [545.5; 1748.9]	1.4225, 0.4910
**Gamma-glutamyl transpeptidase** (GGT), U/L	20.3 [17.5; 24.0]	20.5 [16.8; 25.0]	21.8 [18.2; 25.7]	12.8134, 0.0017
	-	-	*p_1-3_* = 0.0003	
α-amylase, U/L	201.6 [100.5; 404.4]	312.4 [138.0; 514.4]	312.1 [175.2; 650.4]	23.6106, 0.00001
	-	-	*p_1-3_* = 0.0000	
**Antioxidant enzymes**				
Catalase, mcat/L	4.32 [3.20; 5.57]	2.94 [2.24; 4.28]	2.68 [2.02; 4.01]	175.453, 0.00000
	-	*p_1-2_* = 0.0000	*p_1-3_* = 0.0000	
**Superoxide dismutase** (SOD), c.u.	57.9 [34.2; 104.0]	60.5 [34.2; 102.6]	65.8 [29.0; 121.1]	1.4605, 0.4818
**Antioxidant activity** (АОА), mmol/L	2.36 [1.61; 3.48]	2.66 [1.85; 3.24]	2.48 [1.61; 3.62]	2.4126, 0.2993
Peroxidase, c.u.	0.320 [0.170; 0.610]	0.540 [0.300; 1.080]	0.440 [0.240; 0.820]	13.7029, 0.0011
	-	*p_1-2_* = 0.0009	*p_1-3_* = 0.0010	
**Lipoperoxidation Products**				
Diene conjugates, c.u.	3.92 [3.78; 4.06]	3.93 [2.89; 4.13]	3.98 [3.79; 4.16]	14.972, 0.0006
	-	-	*p_1-3_* = 0.0004	
Triene conjugates, c.u.	0.870 [0.793; 0.944]	0.979 [0.843; 1.233]	0.891 [0.787; 1.000]	9.8454, 0.0073
	-	*p_1-2_* = 0.0000	*p_1-3_* = 0.0250	
Schiff bases, c.u.	0.528 [0.492; 0.565]	0.558 [0.495; 0.682]	0.557 [0.489; 0.669]	41.5333, 0.00000
	-	*p_1-2_* = 0.0000	*p_1-3_* = 0.0000	
**Malondialdehyde** (MDA), nmol/mL	6.84 [5.81; 8.38]	7.35 [5.64; 9.32]	7.18 [5.73; 9.49]	5.6906, 0.0581
**Endogenous intoxication rates**				
MM 254 nm, c.u.	0.271 [0.187; 0.381]	0.261 [0.151; 0.407]	0.252 [0.164; 0.398]	3.3012, 0.1919
MM 280 nm, c.u.	0.224 [0.157; 0.324]	0.225 [0.135; 0.359]	0.226 [0.147; 0.348]	0.0782, 0.9617
MM 280/254 nm	0.847 [0.749; 0.948]	0.900 [0.797; 1.004]	0.897 [0.801; 1.011]	28.723, 0.00000
	-	*p_1-2_* = 0.0007	*p_1-3_* = 0.0000	

*p_1-2_*, *p_1-3_*: statistically significant differences compared with control group (Mann-Whitney U-criterion); H: Kruskal–Wallis criterion; *p*: significance level; MM: middle molecules.

**Table 3:** Classification results depending on the stage of lung cancer.

Stage	Correctly Classified Patients	Incorrectly Classified Patients	Sensitivity at the Appropriate Stage, %
T_1-2_N_0_M_0_, *n* = 114	78	37	67.9
T_2_N_1-3_M_0_, *n* = 59	38	21	65.0
T_3_N_0-3_M_0_, *n* = 78	60	18	77.4
T_4_N_0-3_M_0_, *n* = 59	38	21	65.0
T_1-4_N_0-3_M_1_, *n* = 116	84	32	72.2
Total, *n* = 426	298	128	69.5

**Table 4:** Relative risk in the formation of groups relative to medians and interquartile range of IC concentration and LDH activity in saliva.

Feature	Category	Hazard Ratio (95% CI)	*p* Value	Overall Survival, Months
**Median IC and LDH Concentrations**				
IC, mmol/L	<0.311, *n* = 212	1	0.00244	23.0
	>0.311, *n* = 213	1.73 (1.14–2.62) *		14.9
LDH, U/L	<1133, *n* = 212	1	0.04720	15.6
	>1133, *n* = 210	0.72 (0.48–1.09)		21.3
IC, mmol/L + LDH, U/L	<0.311, >1133, *n* = 114	1	0.00000	24.9
	>0.311, <1133, *n* = 116	2.32 (1.29–4.14) *		12.1
**Interquartile Range of** **IC and LDH Concentrations**				
IC, mmol/L	<0.197, *n* = 108	1	0.02742	21.7
	0.197–0.478, *n* = 209	1.41 (0.87–2.28)		17.9
	>0.478, *n* = 108	2.64 (1.42–4.82) *		16.6
LDH, U/L	<545, *n* = 106	1	0.00022	11.5
	545–1748, *n* = 212	0.78 (0.46–1.34)		19.2
	>1748, *n* = 104	0.44 (0.24–0.79) *		23.9
IC, mmol/L + LDH, U/L	<0.197, >1748, *n* = 52	1	0.00000	22.4
	>0.478, <575, *n* = 48	4.17 (1.36–12.51) *		11.7

* differences are statistically significant, *p* < 0.05.

## Supplementary Materials

The following are available online at https://www.mdpi.com/2075-4418/10/4/186/s1, Figure S1: The result of sorting the biochemical parameters of saliva by importance for adenocarcinoma, Figure S2: The result of sorting the biochemical parameters of saliva by importance for squamous cell lung cancer, Figure S3: The result of sorting the biochemical parameters of saliva by importance for neuroendocrine lung tumors, Table S1: Biochemical composition of saliva, depending on the histological type of LC, Table S2: Biochemical composition of saliva depending on the tumor size (T), Table S3: Biochemical composition of saliva depending on lymphogenic metastasis (N).
