# Supplementary material for: Biochemical Markers of Saliva in Lung Cancer: Diagnostic and Prognostic Perspectives
Source: Diagnostics (Basel). 2020 Mar 27;10(4):186. doi: 10.3390/diagnostics10040186 (PMC7235830; doi:10.3390/diagnostics10040186)
Supplement: Supplementary file 1 [file diagnostics-10-00186-s001.pdf]

Supplementary Figures

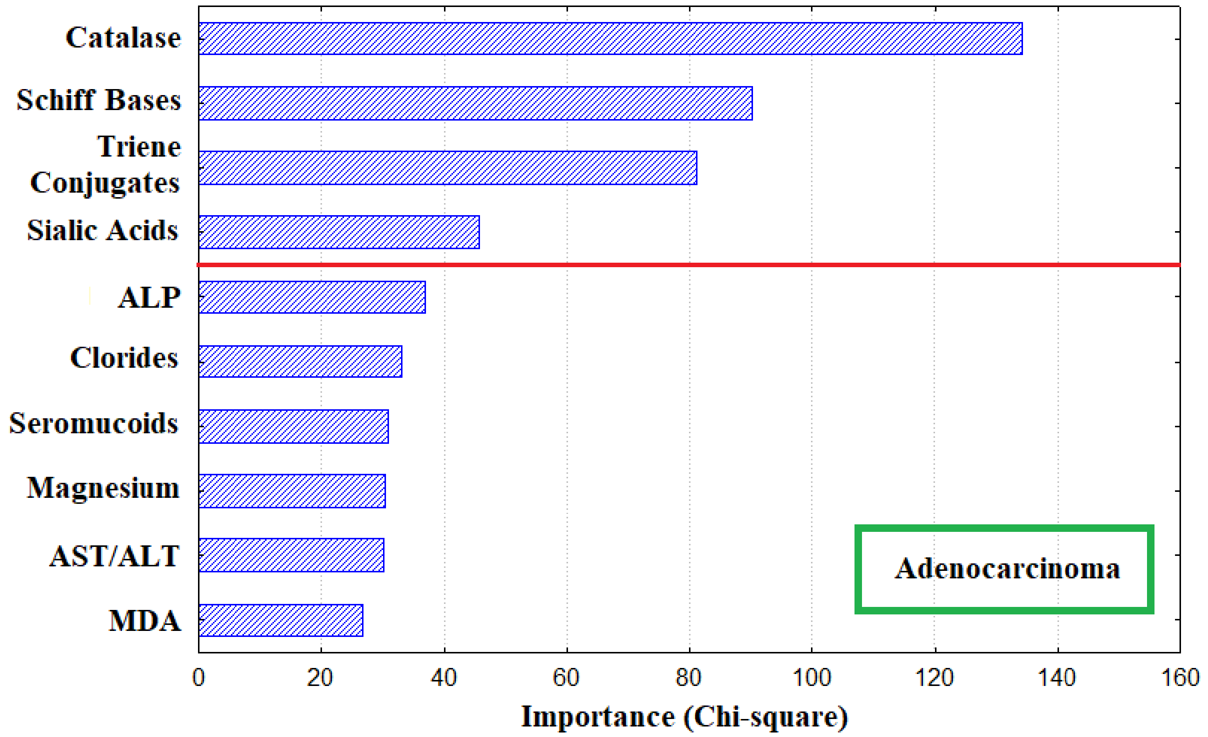

Figure S1. The result of sorting the biochemical parameters of saliva by importance for adenocarcinoma (the first 10 parameters are given). The red line indicates the parameters included in the classifier.

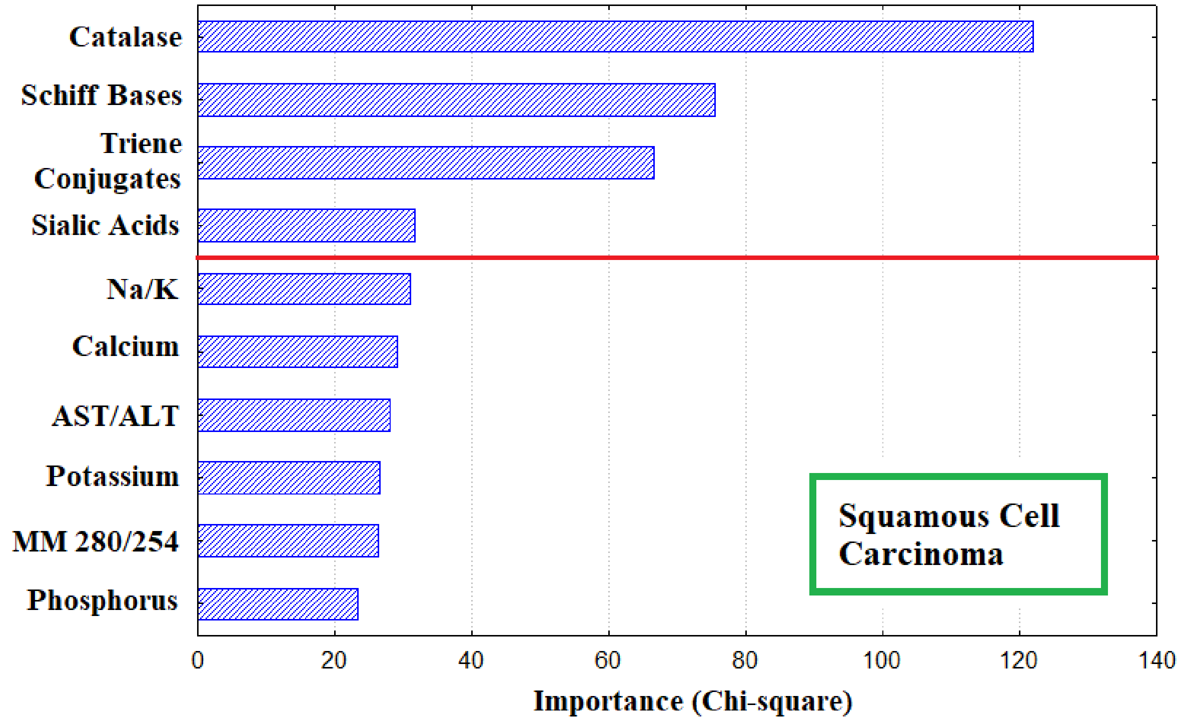

Figure S2. The result of sorting the biochemical parameters of saliva by importance for squamous cell lung cancer (the first 10 parameters are given). The red line indicates the parameters included in the classifier.

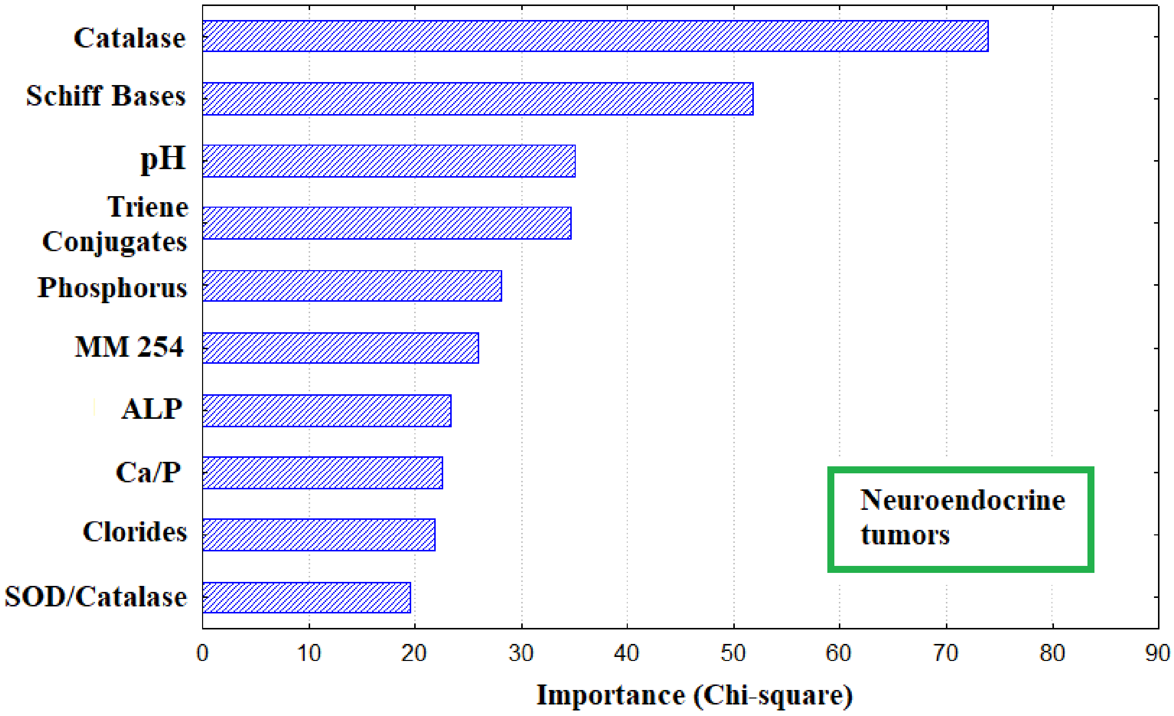

Figure S3. The result of sorting the biochemical parameters of saliva by importance for neuroendocrine lung tumors (the first 10 parameters are given).

Supplementary Tables

Table S1. Biochemical composition of saliva, depending on the histological type of LC.

Indicators	AC, *n* = 189	*p* value	SCC, *n* = 135	*p* value	NEC, *n* = 68	*p* value
**Electrolytes**						
pH	6.49 [6.23; 6.78]	0.5275	6.52 [6.29; 6.80]	0.1843	6.46 [6.15; 6.77]	0.2437
Calcium, mmol/L	1.44 [1.04; 1.86]	0.0493	1.40 [0.97; 1.83]	0.4211	1.33 [1.06; 1.80]	0.5353
Phosphorus, mmol/L	4.33 [3.47; 5.69]	0.1485	4.76 [3.37; 6.06]	0.7957	4.36 [3.03; 5.25]	0.0321
Sodium, mmol/L	9.3 [6.7; 14.6]	0.0720	8.7 [5.4; 12.8]	0.9474	9.2 [6.2; 16.3]	0.1642
Potassium, mmol/L	12.5 [9.3; 15.7]	0.4265	14.1 [11.0; 16.6]	0.0004	12.1 [7.8; 14.3]	0.5565
Chlorides, mmol/L	27.9 [21.5; 36.4]	0.1304	29.5 [22.8; 36.7]	0.0031	27.0 [21.5; 35.7]	0.4429
Magnesium, mmol/L	0.301 [0.230; 0.390]	0.6712	0.294 [0.231; 0.369]	0.5936	0.303 [0.237; 0.349]	0.9402
NO, nmol/mL	23.9 [14.0; 42.3]	0.5833	22.5 [13.7; 41.2]	0.7945	29.7 [17.3; 47.0]	0.0581
**Protein Metabolism**						
Protein, g/L	0.66 [0.35; 1.12]	0.0016	0.65 [0.33; 0.95]	0.0014	0.62 [0.41; 0.98]	0.0171
Albumin, g/L	0.32 [0.16; 0.52]	0.0999	0.30 [0.16; 0.49]	0.3545	0.29 [0.16; 0.40]	0.8655
Urea, mmol/L	8.05 [5.84; 11.31]	0.5729	7.77 [5.42; 11.76]	0.4518	7.93 [5.15; 12.56]	0.7069
Uric acid, nmol/mL	76.73 [34.62; 165.84]	0.5619	103.21 [46.15; 180.69]	0.0096	84.43 [27.35; 148.36]	0.9301
α-amino acids, mmol/L	4.15 [3.85; 4.62]	0.2983	4.16 [3.90; 4.52]	0.2289	4.17 [3.95; 4.68]	0.0908
Imidazole compounds, mmol/L	0.303 [0.197; 0.470]	0.0013	0.296 [0.182; 0.478]	0.0539	0.345 [0.262; 0.505]	0.0002
Seromucoids, c.u.	0.102 [0.055; 0.156]	0.1950	0.104 [0.063; 0.155]	0.1283	0.085 [0.055; 0.156]	0.8035
Sialic acids, mmol/L	0.146 [0.095; 0.262]	0.0000	0.189 [0.101; 0.281]	0.0328	0.201 [0.098; 0.342]	0.6469
**Metabolic enzymes**						
ALT, U/L	3.77 [2.69; 5.54]	0.0755	4.05 [2.85; 5.38]	0.0115	4.23 [2.92; 6.54]	0.0065
AST, U/L	5.17 [3.00; 7.50]	0.3177	5.17 [3.11; 7.00]	0.1117	5.83 [4.20; 9.00]	0.0690
AST/ALT	1.28 [1.01; 1.66]	0.0001	1.20 [0.93; 1.56]	0.0000	1.32 [1.07; 1.75]	0.1109
ALP, U/L	77.14 [45.63; 126.03]	0.0000	71.71 [49.98; 102.13]	0.0010	82.57 [54.33; 124.95]	0.0001
LDH, U/L	1159.5 [598.1; 1730.5]	0.9577	930.7 [498.0; 1831.0]	0.1083	1148.0 [590.8; 1856.0]	0.9764

GGT, U/L	21.6 [18.0; 25.5]	0.0227	22.4 [18.1; 25.7]	0.0068	21.0 [19.2; 25.2]	0.1284
α-amylase, U/L	334.7 [202.3; 659.1]	**0.0001**	252.4 [138.4; 462.2]	0.1425	391.7 [181.1; 716.0]	**0.0019**
**Antioxidant enzymes**						
Catalase, mcat/L	2.66 [2.05; 4.20]	0.0000	2.68 [1.98; 3.65]	0.0000	2.54 [1.84; 3.82]	0.0000
SOD, c.u.	61.8 [23.7; 111.8]	0.7473	60.5 [29.0; 113.2]	0.9836	68.4 [34.2; 126.3]	0.3328
AOA, mmol/l	2.32 [1.59; 3.28]	0.4409	2.53 [1.61; 3.89]	0.7102	2.67 [1.77; 4.42]	0.1973
Peroxidase, c.u.	0.455 [0.340; 0.910]	0.0007	0.430 [0.205; 0.725]	0.2424	0.500 [0.220; 0.840]	0.0448
**Lipoperoxidation Products**						
Diene conjugates, c.u.	3.98 [3.78; 4.18]	0.0045	3.99 [3.82; 4.17]	0.0026	3.98 [3.83; 4.18]	0.0329
Triene conjugates, c.u.	0.894 [0.796; 0.997]	0.0667	0.901 [0.782; 1.002]	0.1453	0.882 [0.773; 1.002]	0.6361
Schiff bases, c.u.	0.551 [0.492; 0.659]	0.0002	0.560 [0.490; 0.664]	0.0000	0.554 [0.485; 0.666]	0.0302
MDA, nmol/ml	7.26 [5.90; 9.44]	0.0298	7.44 [5.56; 9.57]	0.1784	7.01 [5.81; 9.57]	0.2393
**Endogenous intoxication rates**						
MM 254 nm, c.u.	0.258 [0.176; 0.396]	0.7331	0.244 [0.160; 0.395]	0.1014	0.228 [0.154; 0.450]	0.3807
MM 280 nm, c.u.	0.236 [0.154; 0.357]	0.1979	0.219 [0.150; 0.348]	0.8327	0.197 [0.119; 0.368]	0.2733
MM 280/254 nm	0.900 [0.815; 0.997]	0.0001	0.903 [0.804; 1.037]	0.0000	0.873 [0.770; 0.956]	0.3671

p - Differences compared with the control group.

Table S2. Biochemical composition of saliva depending on the tumor size (T).

Indicators	T_1M_0, *n* = 30	T_2M_0, *n* = 153	T_3M_0, *n* = 72	T_4M_0, *n* = 54	$T_{1-4}M_1$, *n* = 116
**Electrolytes**					
pH	6.47 [6.30; 6.71]	6.45 [6.18; 6.69]	6.58 [6.32; 6.82]	6.47 [6.18; 6.88]	6.54 [6.32; 6.88]
Calcium, mmol/L	1.37 [0.92; 1.80]	1.41 [1.03; 1.86]	1.27 [0.93; 1.78]	1.45 [1.08; 1.71]	1.53 [1.05; 1.93]
Phosphorus, mmol/L	4.94 [3.92; 5.85]	4.58 [3.37; 5.78]	4.75 [3.20; 6.08]	4.70 [3.47; 5.80]	3.96 [2.96; 5.36]
Sodium, mmol/L	8.9 [5.5; 13.8]	8.9 [6.1; 14.1]	9.3 [5.2; 13.3]	10.1 [7.1; 17.6]	8.9 [5.0; 13.8]
Potassium, mmol/L	12.9 [10.2; 15.4]	12.7 [9.1; 15.4]	14.2 [11.1; 16.7]	13.0 [9.9; 16.5]	12.7 [8.9; 16.3]
Chlorides, mmol/L	28.4 [23.4; 33.3]	27.4 [22.3; 36.4]	28.7 [21.9; 36.1]	30.5 [22.0; 36.8]	28.6 [21.2; 35.7]
Magnesium, mmol/L	0.313 [0.241; 0.372]	0.313 [0.255; 0.378]	0.279 [0.226; 0.335]	0.306 [0.195; 0.390]	0.292 [0.215; 0.384]
NO, nmol/mL	34.5 [14.0; 52.5]	26.3 [14.7; 41.2]	22.9 [13.5; 41.1]	19.7 [13.5; 44.4]	21.0 [14.2; 40.4]

**Protein Metabolism**					
Protein, g/L	0.74<br>[0.46; 1.04]	0.61<br>[0.31; 1.00]	0.64<br>[0.35; 1.12]	0.72<br>[0.52; 1.13]	0.68<br>[0.29; 1.00]
Albumin, g/L	0.37<br>[0.20; 0.48]	0.31<br>[0.17; 0.46]	0.24<br>[0.15; 0.40]	0.35<br>[0.17; 0.63]	0.27<br>[0.16; 0.50]
Urea, mmol/L	9.08<br>[6.74; 12.33]	8.58<br>[6.41; 12.00]	7.78<br>[6.07; 11.73]	6.96<br>[4.65; 11.63]	7.59<br>[4.93; 11.77]
Uric acid, nmol/mL	81.32<br>[44.23; 238.46]	94.76<br>[47.03; 165.84]	82.19<br>[34.65; 164.60]	89.11<br>[37.13; 176.32]	74.39<br>[26.92; 150.77]
α-amino acids, mmol/L	4.10<br>[3.90; 4.42]	4.19<br>[3.93; 4.69]	4.28<br>[4.03; 4.66]	4.16<br>[3.86; 4.43]	4.07<br>[3.82; 4.53]
Imidazole compounds, mmol/L	0.281<br>[0.197; 0.584]	0.281<br>[0.175; 0.448]	0.281<br>[0.220; 0.417]	0.444<br>[0.250; 0.539]	0.330<br>[0.231; 0.478]
Seromucoids, c.u.	0.136<br>[0.090; 0.166]	0.102<br>[0.064; 0.157]	0.091<br>[0.048; 0.121]	0.087<br>[0.055; 0.160]	0.088<br>[0.046; 0.145]
	-	-	$p = 0.0067$	$p = 0.0432$	$p = 0.0121$
Sialic acids, mmol/L	0.162<br>[0.110; 0.244]	0.192<br>[0.110; 0.305]	0.153<br>[0.085; 0.250]	0.168<br>[0.085; 0.287]	0.171<br>[0.092; 0.272]
**Metabolic enzymes**					
ALT, U/L	4.31<br>[3.31; 5.46]	4.23<br>[2.85; 5.54]	3.58<br>[2.62; 5.69]	3.92<br>[2.62; 5.46]	4.08<br>[2.69; 5.92]
AST, U/L	5.38<br>[3.92; 7.75]	5.58<br>[3.50; 7.92]	5.03<br>[2.67; 6.83]	4.92<br>[2.54; 7.63]	5.17<br>[3.17; 7.67]
AST/ALT	1.19<br>[0.98; 1.53]	1.40<br>[1.07; 1.73]	1.26<br>[0.92; 1.56]	1.23<br>[0.92; 1.58]	1.19<br>[0.89; 1.43]
ALP, U/L	84.75<br>[54.33; 147.76]	79.31<br>[49.98; 117.34]	73.88<br>[48.89; 124.95]	65.19<br>[47.81; 104.30]	70.62<br>[44.55; 110.82]
LDH, U/L	1371.5<br>[549.7; 2093.0]	1206.0<br>[576.6; 1872.0]	970.0<br>[643.3; 1712.5]	979.4<br>[443.6; 1634.0]	1107.0<br>[521.5; 1667.0]
GGT, U/L	23.2<br>[20.8; 28.2]	22.5<br>[18.6; 25.7]	21.3<br>[17.8; 24.5]	22.2<br>[17.4; 27.4]	21.0<br>[18.0; 25.3]
	-	-	$p = 0.0302$	-	$p = 0.0334$
α-amylase, U/L	304.7<br>[121.5; 564.6]	327.7<br>[218.6; 741.5]	249.3<br>[82.5; 421.0]	354.8<br>[231.8; 821.4]	294.2<br>[177.0; 635.2]
**Antioxidant enzymes**					
Catalase, mcat/L	2.94<br>[2.38; 5.41]	2.76<br>[2.05; 4.17]	2.86<br>[2.14; 3.98]	2.56<br>[1.94; 3.48]	2.50<br>[1.88; 3.70]
	-	-	-	$p = 0.0414$	$p = 0.0373$
SOD, c.u.	71.1<br>[40.8; 181.6]	59.2<br>[29.0; 131.6]	65.8<br>[31.6; 128.9]	67.1<br>[19.7; 110.5]	63.2<br>[26.3; 92.1]
AOA, mmol/l	2.05<br>[1.68; 2.85]	2.62<br>[1.71; 4.07]	2.31<br>[0.88; 3.15]	2.88<br>[1.90; 4.22]	1.89<br>[1.47; 3.12]
Peroxidase, c.u.	0.415<br>[0.310; 0.880]	0.460<br>[0.350; 0.930]	0.430<br>[0.300; 0.640]	0.230<br>[0.135; 0.510]	0.430<br>[0.200; 0.660]
**Lipoperoxidation Products**					
Diene conjugates, c.u.	3.94<br>[3.72; 4.31]	3.99<br>[3.80; 4.18]	3.99<br>[3.81; 4.17]	4.01<br>[3.85; 4.15]	3.98<br>[3.77; 4.16]
Triene conjugates, c.u.	0.865<br>[0.813; 0.950]	0.901<br>[0.785; 0.999]	0.877<br>[0.770; 1.000]	0.904<br>[0.808; 1.029]	0.882<br>[0.792; 1.002]

Schiff bases, c.u.	0.503 [0.463; 0.549]	0.560 [0.495; 0.671]	0.565 [0.476; 0.681]	0.570 [0.514; 0.660]	0.559 [0.488; 0.677]
	-	$p = 0.0121$	$p = 0.0384$	$p = 0.0269$	$p = 0.0321$
MDA, nmol/ml	6.97 [5.64; 10.94]	7.26 [5.73; 9.32]	7.65 [5.60; 9.53]	7.56 [5.85; 10.34]	6.75 [5.81; 9.06]
**Endogenous intoxication rates**					
MM 254 nm, c.u.	0.224 [0.192; 0.365]	0.255 [0.159; 0.399]	0.231 [0.165; 0.335]	0.278 [0.149; 0.486]	0.252 [0.155; 0.400]
MM 280 nm, c.u.	0.221 [0.163; 0.336]	0.233 [0.153; 0.369]	0.221 [0.149; 0.308]	0.253 [0.149; 0.436]	0.216 [0.126; 0.362]
MM 280/254 nm	0.904 [0.800; 0.976]	0.895 [0.807; 1.016]	0.901 [0.795; 1.017]	0.940 [0.871; 1.095]	0.864 [0.774; 0.984]

p - Statistically significant differences compared with the T_1M_0 group.

Table S3. Biochemical composition of saliva depending on lymphogenic metastasis (N).

Indicators	N_0M_0, $n = 146$	N_1M_0, $n = 59$	N_2M_0, $n = 88$	N_3M_0, $n = 16$
**Electrolytes**				
pH	6.50 [6.26; 6.72]	6.51 [6.24; 6.80]	6.47 [6.18; 6.74]	6.44 [6.06; 6.68]
Calcium, mmol/L	1.37 [0.98; 1.80]	1.49 [1.08; 1.84]	1.39 [1.00; 1.78]	1.55 [1.06; 1.91]
Phosphorus, mmol/L	4.77 [3.64; 5.91]	4.75 [3.22; 5.72]	4.70 [3.28; 5.88]	3.75 [2.29; 5.83]
Sodium, mmol/L	9.2 [6.4; 13.1]	8.0 [5.7; 10.7]	9.8 [6.1; 17.6]	12.6 [3.2; 17.1]
Potassium, mmol/L	12.9 [10.3; 16.0]	13.2 [9.2; 16.8]	13.2 [9.8; 15.4]	12.5 [6.3; 16.8]
Chlorides, mmol/L	28.2 [22.4; 36.4]	26.0 [21.2; 34.3]	30.3 [23.8; 38.8]	25.8 [20.9; 30.4]
Magnesium, mmol/L	0.309 [0.261; 0.361]	0.307 [0.235; 0.359]	0.306 [0.219; 0.384]	0.287 [0.245; 0.308]
NO, nmol/mL	27.4 [14.2; 46.7]	21.1 [10.9; 41.1]	25.4 [15.0; 40.7]	20.5 [14.4; 32.2]
**Protein Metabolism**				
Protein, g/L	0.63 [0.36; 1.00]	0.78 [0.44; 1.16]	0.64 [0.33; 1.04]	0.59 [0.35; 1.06]
Albumin, g/L	0.33 [0.19; 0.49]	0.30 [0.20; 0.42]	0.31 [0.14; 0.47]	0.22 [0.12; 0.37]
Urea, mmol/L	8.98 [6.43; 13.12]	7.86 [5.78; 11.46]	7.73 [5.45; 11.57]	6.00 [4.31; 10.06]
	-	-	-	$p = 0.0163$
Uric acid, nmol/mL	93.49 [47.03; 172.50]	85.71 [29.70; 160.55]	82.66 [37.72; 167.42]	94.06 [18.41; 223.48]
α-amino acids, mmol/L	4.22 [3.96; 5.00]	4.27 [3.89; 4.66]	4.16 [3.86; 4.45]	4.07 [3.92; 4.30]
Imidazole compounds, mmol/L	0.258 [0.175; 0.448]	0.303 [0.212; 0.455]	0.360 [0.239; 0.539]	0.459 [0.398; 0.558]
	-	-	**$p = 0.0019$**	$p = 0.0069$
Seromucoids, c.u.	0.104 [0.068; 0.168]	0.090 [0.042; 0.124]	0.098 [0.064; 0.159]	0.075 [0.056; 0.123]
	-	**$p = 0.0083$**	-	$p = 0.0410$

Sialic acids, mmol/L	0.177 [0.110; 0.262]	0.150 [0.090; 0.256]	0.171 [0.095; 0.293]	0.201 [0.079; 0.305]
**Metabolic enzymes**				
ALT, U/L	4.08 [3.15; 5.54]	3.91 [2.85; 5.54]	3.92 [2.54; 5.54]	3.85 [2.69; 6.12]
AST, U/L	5.58 [3.58; 7.75]	5.28 [3.00; 7.00]	5.28 [3.08; 7.92]	5.50 [2.54; 7.25]
AST/ALT	1.34 [1.06; 1.73]	1.21 [0.92; 1.56]	1.29 [0.94; 1.66]	1.05 [0.80; 1.54]
ALP, U/L	80.40 [49.98; 130.38]	73.88 [54.33; 121.69]	70.62 [49.98; 111.91]	69.54 [48.89; 110.82]
LDH, U/L	1284.0 [604.7; 1907.0]	1108.0 [649.7; 1824.0]	1039.5 [476.8; 1627.5]	907.6 [402.6; 2261.0]
GGT, U/L	23.1 [19.4; 26.5]	21.2 [17.3; 23.9]	22.1 [17.6; 26.5]	21.6 [18.9; 24.9]
	-	$p = 0.0103$	-	-
α-amylase, U/L	301.6 [138.4; 662.4]	266.5 [78.5; 600.9]	404.5 [265.8; 821.4]	267.6 [147.5; 349.6]
**Antioxidant enzymes**				
Catalase, mcat/L	2.75 [2.17; 4.29]	2.74 [2.06; 4.08]	2.77 [1.98; 3.81]	2.24 [1.72; 3.17]
	-	-	-	$p = 0.0494$
SOD, c.u.	63.2 [26.3; 139.5]	65.8 [36.8; 150.0]	63.2 [29.0; 110.5]	94.7 [36.8; 131.6]
AOA, mmol/L	2.48 [1.70; 3.59]	2.72 [1.50; 4.10]	2.65 [1.77; 4.07]	2.44 [1.05; 5.71]
Peroxidase, c.u.	0.500 [0.370; 0.930]	0.430 [0.260; 0.620]	0.310 [0.175; 0.485]	No data
	-	-	$p = 0.0010$	-
**Lipoperoxidation Products**				
Diene conjugates, c.u.	3.97 [3.76; 4.19]	3.97 [3.80; 4.12]	4.01 [3.84; 4.18]	4.03 [3.79; 4.18]
Triene conjugates, c.u.	0.865 [0.769; 0.962]	0.944 [0.828; 1.007]	0.918 [0.796; 1.023]	0.966 [0.868; 1.078]
	-	$p = 0.0293$	-	$p = 0.0081$
Schiff bases, c.u.	0.528 [0.482; 0.611]	0.591 [0.495; 0.716]	0.582 [0.516; 0.687]	0.662 [0.580; 0.703]
	-	$p = 0.0174$	$p = 0.0024$	$p = 0.0003$
MDA, nmol/mL	7.18 [5.64; 9.96]	6.75 [5.56; 9.06]	7.52 [5.73; 10.00]	8.55 [6.15; 10.77]
**Endogenous intoxication rates**				
MM 254 nm, c.u.	0.255 [0.175; 0.399]	0.239 [0.165; 0.336]	0.257 [0.151; 0.447]	0.218 [0.167; 0.336]
MM 280 nm, c.u.	0.226 [0.154; 0.370]	0.219 [0.148; 0.307]	0.238 [0.138; 0.362]	0.243 [0.193; 0.350]
MM 280/254 nm	0.906 [0.804; 1.016]	0.900 [0.817; 0.982]	0.907 [0.808; 1.060]	1.099 [0.928; 1.355]
	-	-	-	$p = 0.0005$

p - Statistically significant differences compared with the N_0M_0 group.
